# Enterovirus 71 Infection with Central Nervous System Involvement, South Korea

**DOI:** 10.3201/eid1611.100104

**Published:** 2010-11

**Authors:** Wi-Sun Ryu, Byunghak Kang, Jiyoung Hong, Seoyeon Hwang, Ahyoun Kim, Jonghyun Kim, Doo-Sung Cheon

**Affiliations:** Author affiliations: Seoul National University Hospital, Seoul, South Korea (W.-S. Ryu);; Korea Centers for Disease Control and Prevention, Seoul (B. Kang, J. Hong, S. Hwang, A. Kim, D.S. Cheon);; Catholic University College of Medicine, Suwon, South Korea (J. Kim)

**Keywords:** Enterovirus, central nervous system, outbreak, viruses, hand-foot-and-mouth disease, South Korea, dispatch

## Abstract

We assessed neurologic sequelae associated with an enterovirus 71 (EV71) outbreak in South Korea during 2009. Four of 94 patients had high signal intensities at brainstem or cerebellum on magnetic resonance imaging. Two patients died of cardiopulmonary collapse; 2 had severe neurologic sequelae. Severity and case-fatality rates may differ by EV71 genotype or subgenotype.

Several major outbreaks of enterovirus 71 (EV71) have been reported since 1974 (*1*,*2*). Countries of the Asia Pacific Rim particularly have been recently affected by large outbreaks of EV71-associated hand-foot-and-mouth disease (HFMD). Most patients with HFMD experience a mild disease course, but recent reports on the outbreak of EV71 infection in various countries, including Taiwan, People’s Republic of China, and Malaysia, indicate that some EV71-infected persons have severe neurologic complications or systemic disease (*3*,*4*).

The varying prevalences of neurologic complications of EV71 infection among outbreaks are assumed to have been driven by differences of genotypes and co-infection with other viruses, such as a newly characterized adenovirus; however, the exact reasons remain unclear (*3*,*5*,*6*). We report an outbreak of EV71 infection with neurologic involvement on the basis of information from a prospective, clinical, and virologic study that was collected through South Korea’s nationwide surveillance system.

## The Study

The EV surveillance system in South Korea consists of 62 clinics (8 primary clinics, 14 secondary hospitals, and 40 tertiary hospitals located nationwide) managed by pediatric physicians (Figure). During 2009, a total of 2,427 cases of viral disease were reported to the Korea Centers for Disease Control and Prevention through a web-based system. In addition, an experienced neurologist (W.-S.R.) collected detailed clinical information about, and results of imaging studies of, patients reported to have central nervous system (CNS) involvement. We monitored the patients until they were discharged or for 3 weeks if duration of hospitalization was >3 weeks. Patient outcome was classified into 1 of 4 groups; no sequelae (neurologic dysfunction without dependency), mild sequelae, severe sequelae (neurologic dysfunction requiring assistance), or death.

**Figure:**
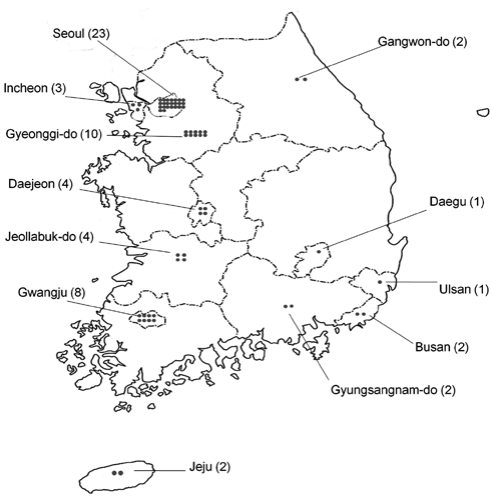
Geographic distribution of clinics participating in enterovirus surveillance, South Korea, 2009.

EV genome detection was attempted by real-time reverse transcription–PCR (RT-PCR) by using TaqMan technology (Applied Biosystems, Foster City, CA, USA). Briefly, viral RNAs were extracted by using the magnetic bead–based viral nucleic acid purification protocol described by Boom et al. (*7*). Subsequently, 1-step real-time RT-PCR was performed by using a dual-labeled fluorogenic EV-specific probe and primers designed on the basis of previous data (*8*). For genotyping, seminested RT-PCR was used to amplify part of the viral protein (VP) 1 gene of EV, based on the Korea Centers for Disease Control and Prevention protocol for detection of pan-EV, and sequencing analysis for VP1 amplicon was performed by using automatic sequencer and DNAstar software package (*9*).

In 2009, a total of 2,427 patients were recruited. Of these patients, 519 had symptoms of HFMD or herpangina. EV was detected in 461 (19%) of all patients and in 321 (66%) of patients with suspected HFMD and herpangina. Samples from 331 (72%) of the 461 EV-seropositive patients were available for genotyping (Table 1). In addition, we found 112 cases of HFMD with CNS complications (meningitis or encephalitis); EV was detected in 95 (85%) and EV71 in 92 (82%) case-patients. Furthermore, EV71 was detected in 2 of the 187 case-patients in which meningitis without HFMD or herpangina was diagnosed. Thus, 94 patients were enrolled in this study. The incidence of EV71 infection peaked in July and decreased drastically in August.

**Table 1:** Genotype distribution of enteroviruses, South Korea

Genotype	No. cases (%)
CA2	24 (7.3)
CA5	35 (10.6)
CA6	32 (9.7)
CA12	3 (0.9)
CA16	29 (8.8)
CB1	8 (2.4)
E3	8 (2.4)
E6	3 (0.9)
E9	2 (0.6)
E11	8 (2.4)
E30	2 (0.6)
E33	3 (0.9)
E71	174 (52.6)
Total	331

Fifty-nine (63%) patients were boys. Mean (SD) patient age was 46 (29) months (range 1 month–12 years); 12 (13%) patients were <1 year of age. Initial diagnoses were viral meningitis (60 patients), encephalitis (20), acute cerebellar ataxia (12), acute transverse myelitis (1), and Guillain-Barré syndrome (1). Rash and fever were the most common initial symptoms (85% and 81%, respectively). Approximately 50% of patients had headache, vomiting, and neck stiffness. Cerebrospinal fluid (CSF) profile was available for 77 patients. Median leukocyte count was 111 cells/mm^3^ (interquartile range 48–318 cells/mm^3^), mean (SD) protein level was 43 (18) mg/dL, and mean (SD) glucose level was 69 (13) mg/dL. Of the 32 (34%) patients who underwent magnetic resonance imaging of the brain, 24 (75%) had normal results, 4 (13%) had meningeal enhancement on T1-enhanced imaging, and 4 (13%) had high signal intensity at the brainstem or cerebellum on T2-weighted or fluid-attenuated inversion recovery imaging. All brain parenchymal lesions were located in the brainstem or cerebellum, and the 4 patients with brain lesions subsequently experienced ataxia.

EV71 was found in 72 (92%) of 78 lower gastrointestinal tract samples, 37 (60%) of 62 upper respiratory tract samples, and 2 (5%) of 37 CSF samples. On the basis of sequence analysis, C4a, with high similarity to strains from China in 2008, was a dominant serotype of EV71 (76%); and C1 was found in 2 patients and C5 in 1 patient.

The results of this and previous studies are summarized in Table 2. The partial VP1 sequences of Korean EV71 strains were registered in GenBank (HM443164–644), and viral genetic identity belonged to C4a genotype, which was not a prevailing genotype in the previous reports of other Asian Pacific countries.

**Table 2:** Enterovirus 71 outbreaks, Asia*

Reference	Outbreak location, year	HFMD			Complicated HFMD		Genotypes detected	GenBank accession nos.
		No. patients	No. patients with EV71		No. patients	No. patients with EV71		
Zhang et al. (*10*)	Fuyang, PRC, 2008	151	59		112	42	C4a	EU703812–14, GQ121417–41
Zhang et al. (*11*)	Shandong, PRC, 2007	105	55		11	6	C4a	EU753365–417
AbuBakar et al. (*1*)	Brunei, 2006	>100	34		NA	NA	B4, B5	FM201328–61
Ooi et al. (*12*)	Sarawak, Malaysia, 2000–2004	773	277		102	56	B4, C1, B5	AY905549–50, AY794036, AF376069
Lin et al. (*13*)	Taiwan, 1998	NA	NA		405	78	C2	AY055194–97, FJ357343
This study	South Korea, 2009	519	168		112	92	C4a, C1, C5	HM443164–644

*HFMD, hand-foot-and-mouth disease; EV71, enterovirus 71; PRC, People’s Republic of China; NA, not available.

## Conclusions

The severity of, and case-fatality rate for, EV71 infection in our population were relatively low compared with those of previous reports in which the case-fatality rate ranged from 10% to 26% (*12*). This discrepancy has some plausible explanations. In previous studies, the prevailing genotype of EV71 was B (*1*,*14*). In contrast, the predominant genotype in our patients was C4, particularly C4a, which has been prevalent in China since 2008 (*11*). The case-fatality rate also was low in China, with 3 patients dying of the 1,149 reported with EV71 infection (*11*). Therefore, the virulence of the C4a genotype may be milder than that of other genotypes.

We detected EV71 rarely in the CSF of our patients. Possible explanations include the transient presence of the virus in CSF, a lower viral load in CSF, and use of an EV PCR assay that had not been optimized to detect EV71. In our population, the CSF profile of EV71 infection appeared to be broadly similar to that of other cases of viral encephalitis or meningitis. Thus, analyzing the CSF of patients with suspected EV71 infection may provide minimal information.

Several studies have shown that EV71 infection rate was most common during the warmer season (*15*). In our study, the seasonality of EV71 infection initially was similar to that of previous reports. However, the prevalence of EV71 infection decreased drastically in August, the warmest month in South Korea. A possible reason for this difference could be that in 2009, influenza pandemic (H1N1) 2009 affected South Korea; with the first death caused by it in South Korea reported in August. As a consequence, personal hygiene practices, such as handwashing and covering one’s cough or sneeze, were emphasized to prevent virus spread. Considering the transmission route of EV71 infection, the emphasis on personal hygiene may thus have hindered the spread of EV71, as well as of the influenza virus.

We report 94 cases of PCR-confirmed EV71 infection with CNS involvement, including 2 deaths, and provide additional clinical and virologic information about EV71. We confirmed that EV71 commonly involved the brainstem and cerebellum, and therefore ataxia is not uncommon in EV71 infection with CNS involvement. In addition, our study supports the hypothesis that the severity of and case-fatality rates for EV71 infection may differ by genotype or subgenotype of EV71.
